# A randomized controlled trial reporting functional outcomes of cognitive–behavioural therapy in medication-treated adults with ADHD and comorbid psychopathology

**DOI:** 10.1007/s00406-016-0735-0

**Published:** 2016-10-17

**Authors:** Susan Young, Brynjar Emilsson, Jon Fridrik Sigurdsson, Mizanur Khondoker, Florence Philipp-Wiegmann, Gisli Baldursson, Halldora Olafsdottir, Gisli Gudjonsson

**Affiliations:** 1 Centre for Psychiatry, Imperial College London, Du Cane Road, London, W12 0NN UK; 2 Broadmoor Hospital, WLMHT, Crowthorne, UK; 3 Reykjavik University, Reykjavik, Iceland; 4 Institute of Psychiatry Psychology and Neuroscience, King’s College London, London, UK; 5 Landspitali -The National University Hospital of Iceland, Reykjavik, Iceland; 6 University of Iceland, Reykjavik, Iceland; 7 Department of Applied Health Research, University College London, London, UK

**Keywords:** ADHD, RCT, Treatment, Reasoning and rehabilitation, R&R2, Cognitive behaviour therapy

## Abstract

Studies assessing psychological treatment of attention deficit hyperactivity disorder (ADHD) in adults are increasingly reported. However, functional outcomes are often neglected in favour of symptom outcomes. We investigated functional outcomes in 95 adults with ADHD who were already treated with medication and randomized to receive treatment as usual (TAU/MED) or psychological treatment (CBT/MED) using a cognitive–behavioural programme, R&R2ADHD, which employs both group and individual modalities. RATE-S functional outcomes associated with ADHD symptoms, social functioning, emotional control and antisocial behaviour were given at baseline, end of treatment and three-month follow-up. The Total composite score of these scales is associated with life satisfaction. In addition, independent evaluator ratings of clinicians who were blind to treatment arm were obtained on the Clinical Global Impression scale at each time point. CBT/MED showed overall (combined outcome at end of treatment and 3-month follow-up) significantly greater functional improvement on all scales. Post-group treatment effects were maintained at follow-up with the exception of emotional control and the Total composite scales, which continued to improve. The largest treatment effect was for the RATE-S Total composite scale, associated with life satisfaction. CGI significantly correlated with all outcomes except for social functioning scale at follow-up. The study provides further evidence for the effectiveness of R&R2ADHD and demonstrates the importance of measuring functional outcomes. The key mechanism associated with improved functional outcomes is likely to be behavioural control.

## Introduction

Attention deficit/hyperactivity disorder (ADHD) is a common disorder of childhood that, for many, persists into adulthood [1]. The disorder is characterized by core symptoms of inattention, hyperactivity and/or impulsivity defined in DSM-5 [2] and ICD-10 [3]. Those diagnosed with the disorder display impairments in their personal and social functioning (e.g. educational attainment, occupational difficulties and relationship problems) [4].

ADHD may be lifelong disorder that has a profound effect on an individual’s quality of life. Biederman et al. [5] conducted a 16-year follow-up of 140 boys with ADHD into their thirties and compared them with 120 boys without ADHD of a similar age. They found that the ADHD cohort had greater impairment in social functioning and daily living. In particular, they had greater family conflict, dependence on parents for financial support and lower socio-economic status. The risk of impaired functional outcomes seems to be considerably greater for young people with ADHD who do not receive treatment. By analysing the data derived from 351 studies, Shaw et al. [6] reported that, compared to people without ADHD, 74 % of functional outcomes were worse for people with untreated ADHD. However, with treatment, 72 % of functional outcomes improve over the longer term. Domains of self-esteem, social function, academic performance and antisocial behaviour may respond particularly well to multimodal treatments that combine both pharmacological and non-pharmacological modalities [4].

A range of intervention strategies is available to treat children and adults with ADHD, including psychological and pharmacological interventions. International guidelines recommend a multimodal treatment approach comprising both pharmacological and psychological interventions [7], and there is empirical support for a larger treatment effect for functional outcomes when using a combined approach over the longer term [4]. Psychological treatments in adults have generally been based on cognitive–behavioural therapy (CBT) [8]. Group interventions are attractive for clinical delivery as they are cost-effective as well as demonstrating medium to large treatment effects for the reduction in ADHD symptoms in randomized control trials of medicated ADHD patients [9–15]. However, most studies typically focus on clinical outcomes rather than functional outcomes as primary measures of success, yet the latter are important markers of treatment efficacy due to their translational value, i.e. because they often relate to functional activities of daily living.

Young and Gudjonsson [16] demonstrated that functional impairments associated with neuropsychological test scores, clinical symptoms of anxiety and depression, and psychosocial performance are all significantly related to underlying ADHD symptoms but, for some individuals, these impairments improve with remission of symptoms. However, for others, residual problems will persist with patients seeking psychiatric help in adulthood. Gudjonsson et al. [17] investigated the relationship between satisfaction with life, ADHD symptoms and associated functional problems measured by the RATE-S (emotional, social and antisocial) among young people in the community. ADHD symptoms and associated problems were significantly related to less satisfaction with life. Poor social functioning was the single best predictor of dissatisfaction with life in males, whereas in females it was poor emotional control. The study shows that even in samples where only mild ADHD symptoms were identified, satisfaction with life is adversely affected. The Total RATE-S scale was overall the best predictor of satisfaction with life.

A study conducted by Emilsson et al. [12] reported significant and large treatment effects at 3-month follow-up for functional outcomes (assessed by the RATE-S scales) for each of its four subscales (ADHD symptoms, emotional control, antisocial behaviour and social functioning) and the total score. However, their study only involved 54 participants, 27 in each group, and there was a substantial amount of missing data at the end of treatment and at three-month follow-up for both the CBT/MED and TAU/MED groups which may have led to a biased estimate of the treatment effect. To reduce possible bias, White et al. [18] recommend analysing all the observed outcome data via the maximum likelihood method under missing data random (MAR) assumption. This requires the inclusion of any relevant predictors of missing data in the analysis model.

The present study aimed to investigate functional outcomes of a multimodal treatment provided to adults with ADHD who were receiving medication and were randomized to receive CBT/MED or treatment as usual (TAU/MED). We have previously reported clinical outcomes [15], and in this study we report functional outcomes using the RATE-S scales [19] which were developed to assess attention, social functioning, emotional control and antisocial behaviour. Outcomes were assessed post-group and at three-month follow-up. The RATE-S Total composite scale is associated with satisfaction [17]. We performed an intention-to-treat analysis using a linear mixed model and analysed for three possible predictors of missing data: gender, age and antisocial personality traits. Thus, we analysed the effects of treatment over time (i.e. end of treatment versus at three-month follow-up) as well as overall group differences in the outcome measures whilst controlling for possible group imbalances caused by missing data.

It was hypothesized that the CBT/MED group would show significantly greater functional improvement compared with the TAU/MED group (after adjusting for missing data and possible confounders) in the RATE-S Total composite scale and across each of the four RATE-S scales (ADHD symptoms, emotional control, antisocial behaviour and social functioning). Second, treatment gains were expected to be maintained at three-month follow-up. Third, it was hypothesized that functional outcomes on the RATE-S would correlate with independent evaluator ratings of the Clinical Global Impression assessment of illness severity.

## Method

### Trial design

This study has been described in detail in our previous study reporting clinical outcomes [15]. Briefly, a parallel-group RCT was conducted at an ADHD outpatient setting within the Mental Health Services at Landspitali—The National University Hospital of Iceland. All participants meeting inclusion criteria were independently and individually randomly allocated (1:1) to receive the R&R2ADHD programme (CBT/MED) or treatment as usual (TAU/MED). Assessments occurred at three time points: baseline, end of treatment and 3 months after treatment. The study was registered with the international clinical trials registry (ACTRN12611000533998).

### Participants

Participants were outpatients at the Mental Health Services at the Landspitali University Hospital, referrals from private practitioners or self-referrals from an advertisement placed with a national ADHD support group (Icelandic ADHD Association). Participants were over 18 years of age, had a current ADHD diagnosis and reported they had remained stable on prescribed ADHD medication for at least 1 month. It was requested that the participants kept their medication dosages unchanged during the study. Exclusion criteria included severe mental illness (i.e. psychotic disorders, bipolar disorder), active suicidal ideation, severe eating disorder, history of drug abuse and general intellectual impairment as without modification the treatment programme would not be suitable for these patient groups. Exclusion criteria were evaluated from a review of the patient’s medical record and a baseline assessment by an experienced mental health practitioner (see baseline assessments section).

**Fig. 1:**
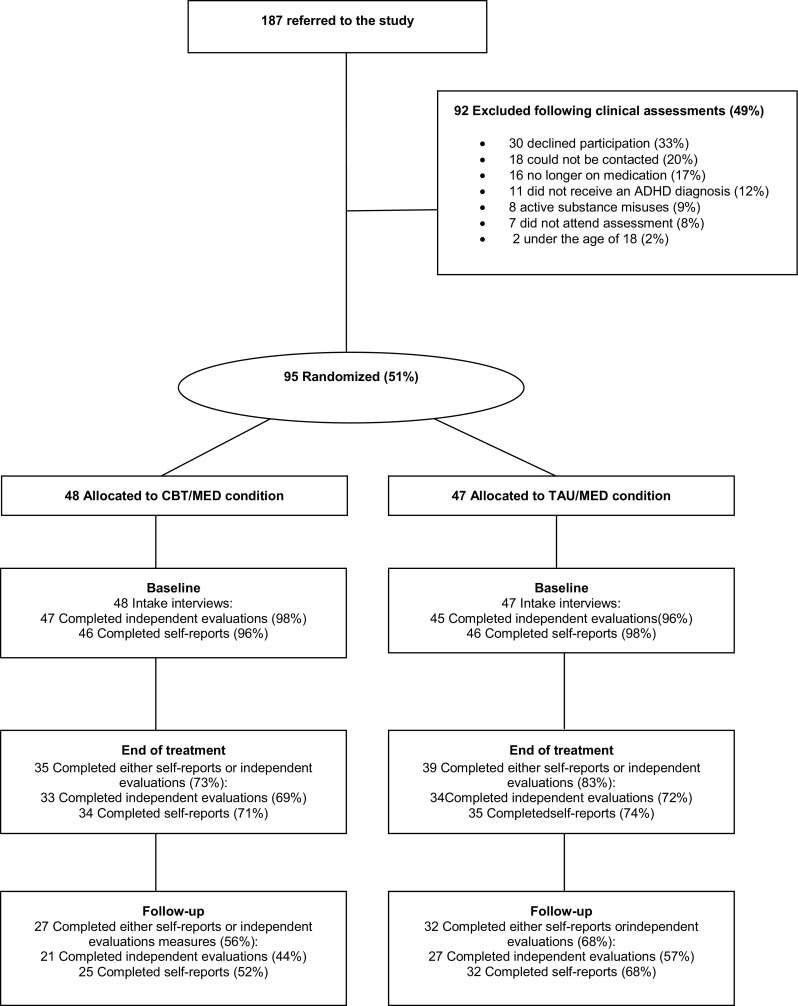
Flow chart of patient participation

A total of 187 patients were referred and out of those 95 (51 %) participated in the study. Figure 1 provides the reasons for non-participation. Eleven participants were excluded because at the intake interview they did not meet DSM-IV diagnostic criteria; 62 (65.3 %) of the participants were female (mean age = 35.00, SD = 11.81); and 33 (34.7 %) were male (mean age = 35.45, SD = 11.62).

Table 1 gives the demographic and clinical characteristics of the study sample. Psychiatrists prescribed medication at baseline; 79 (83.2 %) were prescribed methylphenidate; and 16 (16.8 %) atomoxetine. Five participants were also taking bupropion. In addition, 63 (66.3 %) participants were taking other prescribed medications including antidepressants, benzodiazepines, insulin and ibuprofen.

**Table 1:** Demographic, clinical and baseline characteristics of the study sample (*n* = 95)

	Total *n* = 95	CBT/MED *n* = 48	TAU/MED *n* = 47	Statistics
Gender and age				
Men Women Age Age range	33 (43.7 %) 62 (65.3 %) *M* = 35.17 (SD = 11.68) 18–73	18 (37.5 %) 30 (62.5 %) *M* = 34.19 (SD = 10.58) 18–68	15 (31.9 %) 32 (68.1 %) *M* = 36.17 (SD = 12.75) 18–73	*χ* ^2^ = 0.327; *p* = 0.57 *t*(93) = − 0.826; *p* = 0.41
Marital status				
Single In relationship	47 (49.5 %) 47 (49.5 %)	23 (48.9 %) 24 (51.1 %)	24 (51.1 %) 23 (48.9 %)	*χ* ^2^ = 0.189; *p* = 0.66
Employment status				
Employed Training Pension/unemployed	41 (43.2 %) 22 (23.2 %) 32 (33.7 %)	18 (37.5 %) 14 (29.2 %) 16 (33.3 %)	23 (48.9 %) 8 (17.0 %) 16 (34.0 %)	*χ* ^2^ = 2.236; *p* = 0.33
Medical history				
History of serious illness History of head trauma History of serious accidents History of receiving psychotherapy	27 (28.4 %) 36 (37.9 %) 33 (34.7 %) 69 (72.6 %)	13 (27.1 %) 19 (39.6 %) 17 (35.4 %) 33 (68.8 %)	14 (29.8 %) 17 (36.2 %) 16 (34.0 %) 36 (76.6 %)	*χ* ^2^ = 0.085; *p* = 0.77 *χ* ^2^ = 0.118; *p* = 0.73 *χ* ^2^ = 0.020; *p* = 0.89 *χ* ^2^ = 0.735; *p* = 0.39
ADHD-specific medication				
Methylphenidate Atomoxetine Bupropion Other medications*	73 (83.2 %) 16 (16.8 %) 5 (5.3 %) 63 (66.3 %)	40 (83.3 %) 8 (16.7 %) 3 (6.3 %) 32 (66.7 %)	33 (83.0 %) 8 (17.0 %) 2 (4.3 %) 31 (66.0 %)	*χ* ^2^ = 0.002; *p* = 0.963 *χ* ^2^ = 0.002; *p* = 0.963 *χ* ^2^ = 0.234; *p* = 0.629 *χ* ^2^ = 0.005; *p* = 0.942

* Other medications include for example antidepressants, benzodiazepines, insulin and ibuprofen

In addition to ADHD, participants reported comorbid depression (63.2 %), anxiety (36.8 %) and history of drug/alcohol abuse (15.8 %). Seven participants (7.4 %) reported that they had been diagnosed with a personality disorder and four (4.2 %) with Asperger's syndrome in childhood. Four (4.2 %) reported having post-traumatic stress disorder (PTSD) and two (2.1 %) with a history of eating disorder.

### Interventions

R&R2ADHD is a CBT intervention programme developed for youth and adults with ADHD [20]. It is a revised version of the 35-session Reasoning & Rehabilitation prosocial competence training programme which has a strong evidence base [21]. It was revised to be a shorter and more relevant intervention for individuals presenting with symptoms associated with ADHD. The revision, R&R2ADHD, is a structured, manualized programme consisting of fifteen 90-minute sessions (excluding a mid-session break) five treatment modules: (a) neurocognitive, e.g. learning strategies to improve attentional control, memory, impulse control and planning; (b) problem solving, e.g. developing skilled thinking, problem identification, consequential thinking, managing conflict and making choices; (c) emotional control, e.g. managing feelings of anger and anxiety; (d) prosocial skills, e.g. recognition of the thoughts and feeling of others, empathy, negotiation skills and conflict resolution; and (e) critical reasoning, e.g. evaluating options and effective behavioural skills. R&R2ADHD is a group treatment supplemented by one-to-one meetings with a mentor. In the present study, the group sessions were delivered twice per week (i.e. with a total duration of 8 weeks). The mentors met with the participants between each group session in order to support participants to transfer skills learned in the group into their daily lives. Programme integrity was ensured by regular supervision from the programme author (SY). Furthermore group sessions were delivered according to the manual by experienced CBT therapists who had received training and accreditation to deliver the programme. The mentoring sessions were provided by psychology students who also received training, supervision and written guidance.

*Treatment as usual* was classified as receiving usual treatment, which included both pharmacological and non-pharmacological treatments. All participants received ADHD medication, but non-pharmacological interventions were not systematically recorded.

### Measures

#### Baseline assessments

All of those referrals who could be contacted and who consented to participate in the study were interviewed by an experienced mental health practitioner prior to randomization to ascertain clinical diagnosis according to DSM-IV criteria using the MINI International Neuropsychiatric Interview [22]. In addition, the 54-item Gough Socialization Scale [23] was used to measure antisocial personality (ASP) traits. ASP traits have been commonly found in outpatients diagnosed with ADHD [24] and have been associated with failure to attend follow-up appointments after treatment and hence may relate to missing follow-up data [25]. Socio-demographic data and medical information from a review of clinical records were obtained (see Table 1). IQ scores were not systematically recorded in the clinical records, but individuals were excluded if reference was made to general intellectual impairment. In addition, a battery of measures that were completed by either self-report or by the independent evaluator was administered. Those relating to the present study are:

1. The RATE-S [19] is a revision of the YAQ-S scale [26] representing the 8 items with the highest factor loading from each of the four YAQ-S subscales. It has 32 self-reported items relating to functioning and behaviour in the past month. It consists of four subscales: (1) ADHD symptoms (i.e. items relating to attentional difficulties, impulsiveness and disorganization), (2) emotional control (i.e. items relating to emotional volatility and include worries, anxieties, depressed mood, anger, loss of temper and poor self-esteem), (3) antisocial behaviour (i.e. involvement in a range of delinquent behaviours’ such as fighting, theft, damage to property, vandalism, reckless behaviour, verbal threats to others, and being arrested and questioned by police), and (4) social functioning (i.e. items focus on social participation and confidence in social activities). Behaviours during the previous month are rated on an 8-point scale, ranging from “not at all” to “most of the time”. All scales have shown good internal consistency, as measured by Cronbach’s alpha (i.e. exceeding 0.80 both for people with ADHD and for normal controls). The scales also demonstrate a good discrimination between ADHD patients and clinical controls [26] and a good construct validity, both for patients and for controls [24]. In the Icelandic translation of the RATE-S scale, the measure has demonstrated good reliability and validity [17, 27].
2. Clinical Global Impression [28] consists of a single-question observer rating of severity of illness on a 7-point scale. It focuses on judgment regarding impairment in functioning, symptom severity and distress or coping and is supported by examples of these factors. Clinicians who were blind to treatment condition completed the CGI, which has been widely used in treatment evaluation studies and found to correlate with ADHD severity measured by the adult ADHD investigator symptom rating scale [29].

### Procedure

A battery of self-rated and clinician-rated evaluations (the latter being blind to treatment allocation) was completed at the three time points (baseline, post-treatment and three-month follow-up). Participants were randomized to either the CBT/MED or TAU/MED condition by a psychiatrist at Landspitali University Hospital, who was not involved in the study. The psychiatrist did not posses information about the participants and had received numbers that were pre-assigned to the participants. Block randomization was performed at the time of each study phase by using equal block sizes. The researchers only received the final randomization numbers to protect the concealment of the allocation as proposed in various studies [30, 31]. The R&R2ADHD programme was delivered twice per week by experienced CBT therapists who had received training to deliver the programme. Group participants met their mentor for at least 30 min between group sessions. Mentors had attended a training session to fulfil this role, which involved an introduction to the programme and the mentoring role. In addition, mentors had a manual that guided them through topics to be discussed within the mentoring session. They were provided with supervision once a fortnight from the lead group therapist. There were a total of five R&R2ADHD treatment groups. Participants in the TAU/MED condition received pharmacological intervention and some non-pharmacological interventions, but these were not systematically provided or recorded.

### Statistical analyses

The statistical analysis first involved applying a logistic regression model to identify factors (i.e. age, gender and antisocial personality traits) that might predict the probability of missing data. Only age was significantly associated with the probability of dropouts (i.e. younger participants more often failed to attend the assessment interviews with the independent raters (CGI, *Z* = −2.35, *p* = 0.019).

An intention-to-treat analysis (i.e. individuals analysed in the group to which they were randomized to) of available outcome data was subsequently performed to estimate the effect of offering the treatment using a linear mixed model whilst controlling for age. The random component of the mixed model included random intercept term for participant identifier to take account of between participant variability and the correlation between the repeated measures. Within the fixed part of the model, the treatment effect was adjusted for time (i.e. a binary indicator of whether an outcome measure corresponds to follow-up or end of treatment) and the baseline measures of the respective outcome in all models. We also tested condition by time interactions, but none were found statistically significant and therefore were excluded from the model.

There was minimal amount of missing data at baseline, but there was a substantial proportion (i.e. 37 % and 48 % for the RATE-S and CGI, respectively) of missing data in the completion of the outcome measures due to study dropouts. The dropout rate was similar for CBT/MED and TAU/MED groups (see Fig. 1) with *χ* ^2^ tests showing no significant differences between groups.

Two approaches are typically recommended for dealing with the risk of potential bias due to missing data: the multiple imputation and complete case analysis via maximum likelihood. Since missing data only occurred in the outcome variables, the complete case analysis via maximum likelihood was the most appropriate method. We therefore adopted the complete case analysis approach under a missing at random (MAR) assumption. Covariates predicting missingness were identified using a logistic regression analysis, and an analysis of all observed outcome data was performed using linear mixed model via maximum likelihood method controlling for predictors of missing data, which should produce unbiased estimates under MAR assumption [18].

Adjusted effect sizes, using Cohen’s d, were obtained by calculating the residuals from the respective linear mixed model with the condition term excluded and then calculating the standardized mean difference of the adjusted outcome (residuals) between groups. This calculation was conducted using the user contributed Stata module COHEND [32], which adjusts for uneven group sizes. Table 1 provides descriptive characteristics of the demographic and clinical sample data as well as the outcome measures presented in the form of means and standard deviations. To detect differences between CBT/MED and TAU/MED condition at baseline, independent sample *t* tests were performed and *χ* ^2^ tests were used to analyse categories and categorical data, respectively.

No significant differences were found between the CBT/MED and the TAU/MED groups in the demographic background data.

## Results

### Baseline characteristics

Table 2 shows that there were no significant differences between the CBT/MED and the TAU/MED groups in the baseline outcome measures regarding the RATE-S and CGI.

**Table 2:** Outcome measures in the CBT/MED and TAU/MED conditions and statistics of the baseline measurements

Outcome	CBT/MED			TAU/MED			Statistics of the baseline measurements between groups
	Baseline mean (SD)	End of treatment mean (SD)	Follow-up mean (SD)	Baseline mean (SD)	End of treatment mean (SD)	Follow-up mean (SD)	
RATE Total score	116.60 (28.63) *n* = 45	99.52 (25.77) *n* = 33	87.36 (25.16) *n* = 25	119.28 (27.96) *n* = 46	117.11 (25.46) *n* = 35	115.16 (28.38) *n* = 32	*t*(89) = − 0.452, *p* = 0.652
RATE ADHD symptoms	41.76 (11.05) *n* = 45	34.30 (10.30) *n* = 33	29.28 (11.47) *n* = 25	40.39 (12.05) *n* = 46	38.77 (11.56) *n* = 35	38.66 (11.93) *n* = 32	*t*(89) = 0.563, *p* = 0.575
RATE Emotional control	33.16 (14.12) *n* = 45	27.67 (10.92) *n* = 33	22.84 (9.16) *n* = 25	34.59 (12.34) *n* = 46	31.97 (12.54) *n* = 35	31.38 (12.93) *n* = 32	t(89) = − 0.515, *p* = 0.608
RATE Antisocial scale	11.27 (3.85) *n* = 45	9.24 (1.52) *n* = 33	8.76 (1.67) *n* = 25	12.28 (6.27) *n* = 46	10.29 (2.38) *n* = 35	11.19 (4.03) *n* = 32	*t*(89) = − 0.929, *p* = 0.355
RATE Social functioning	30.42 (8.43) *n* = 45	28.30 (9.19) *n* = 33	26.48 (8.20) *n* = 25	32.02 (9.35) *n* = 46	36.09 (10.44) *n* = 35	33.94 (10.08) *n* = 32	*t*(89) = − 0.857, *p* = 0.394
Clinical global impression	3.96 (0.81) *n* = 47	3.03 (1.05) *n* = 33	3.14 (0.79) *n* = 21	3.91 (1.10) *n* = 45	3.79 (0.77) *n* = 34	3.80 (0.96) *n* = 27	*t*(90) = 0.231, *p* = 0.818

### Outcomes

Table 3 provides an output from the linear mixed model analyses for the RATE-S. Each row shows the coefficient of the treatment indicator (0 = TAU/MED, 1 = CBT/MED) and the relevant inferential statistics of the named outcome variable. All models included a random intercept term for subject identification and controlled for age of participants, time of outcome (indicator of whether the measurement corresponds to end of treatment or follow-up or end of treatment) and the baseline measurement differences of each respective outcome variable. Estimates are provided of the adjusted overall mean differences (i.e. combining the scores from end of treatment and at three-month follow-up between the CBT/MED and TAU/MED groups and the corresponding *p*-values).

**Table 3:** Estimated treatment effect from the linear mixed model analyses with adjusted effect sizes (Cohen’s d) from the model

Outcome	Coef.(*β*)	Standard error	*p* value	95 % CI	Effect size (*d*)
RATE-S Total Score	−16.98	4.04	<0.001	(−24.90, − 9.06)	0.54
RATE-S ADHD symptoms	−5.64	1.58	<0.001	(−8.75, − 2.53)	0.55
RATE-S Emotional control	−4.61	1.92	0.017	(−8.38, − 0.84)	0.32
RATE-S Antisocial scale	−1.4	0.43	0.001	(−2.24, − 0.56)	0.50
RATE-S Social functioning	−5.31	1.48	<0.001	(−8.21, − 2.41)	0.41

There was a significant main effect for all the RATE-S outcomes, suggesting that the CBT group had significantly reduced scores compared to the TAU group at end of treatment. Significant differences emerged between groups on all the outcome variables with low (emotional control and social functioning) to medium (Total, ADHD symptoms, antisocial behaviour) effect sizes.

There was an overall effect of time (end of treatment versus three-month follow-up) adjusted for baseline, group and age, for the emotional control scale (*Z* = −2.01, *p* = 0.04, *d* = 0.32) and the Total scale (*Z* = −2.19, *p* = 0.028, *d* = 0.54) showing steady improvement over time in the treatment group.

Table 4 shows that there was a significant correlation between the CGI and RATE-S scores at baseline, end of treatment and follow-up, mostly with medium to large effects sizes, with two exceptions. The Antisocial scale did not correlate significantly with the CGI scale at the end of treatment; nor did social functioning at follow-up. The correlations were overall most marked for the RATE-S Total scale.

**Table 4:** Correlations between the Clinical Global Impression (CGI) scale at three time periods with the relevant RATE-S scores

Outcome	CGI Baseline	CGI End of treatment	CGI Follow-up
RATE-S Total score	0.46*** *n* = 88	0.52*** *n* = 61	0.54*** *n* = 46
RATE-S ADHD symptoms	0.41*** *n* = 88	0.50*** *n* = 61	0.51*** *n* = 46
RATE-S Emotional control	0.31** *n* = 88	0.33** *n* = 61	0.43** *n* = 46
RATE-S Antisocial scale	0.31** *n* = 88	0.13 *n* = 61	0.43** *n* = 46
RATE-S Social functioning	0.29** *n* = 88	0.43** *n* = 61	0.23 *n* = 46

**p < 0.01, ***p < 0.001

## Discussion

Arnold et al. [4] emphasize the importance of investigating improvements in the personal and social functioning of people treated for ADHD rather than merely focusing on changes in their core symptoms. This was the primary aim of the current study: investigating the functional outcomes (ADHD symptoms, social functioning, emotional control, antisocial behaviour and a general composite Total Scale) of a multimodal treatment provided to adults with ADHD (R&R2ADHD) who were receiving medication and randomized to receive CBT/MED or treatment as usual. The study employed a linear mixed model to control for confounders associated with missing data, between subject variability and the correlation between the measures over time. The treatment effect in the model was adjusted for time, which allowed us to investigate whether or not the treatment effectiveness noted at the end of treatment was maintained or improved at three-month follow-up, in addition to investigating the overall effect of the end of treatment and three-month follow-up combined.

As hypothesized, the CBT/MED group showed an overall significant treatment effect (i.e. the RATE-S Total composite scale) with a medium effect size. In addition, there were significant effects on all four subscales, with medium effect sizes being noted for the ADHD and Antisocial scales. Hence, the perception of group participants was that they had experienced significantly greater functional improvement at the end of the group treatment compared with those receiving TAU. Importantly, the findings show that there were both significant group effects and time effects with the treatment effect being maintained at three-month follow-up for all scales except for emotional control and Total composite scales which continued to improve after the group had ended. This supports previous findings that anxiety, depression and quality of life also continue to improve over time following group completion [15] and extends those findings by showing that clinical improvements translate into daily activities and behaviours. Future research should include functional outcomes that are assessed at follow-up as well as post-treatment in order to capture some benefits of treatment that may present later due to the apparent time lag for some outcomes.

The hypothesis that functional outcomes on the RATE-S would correlate with independent evaluator ratings of the CGI assessment of illness severity was supported at baseline, end of treatment and follow-up with the exception of the social functioning scale at follow-up. Large effect size correlations were found between the CGI and the RATE-S Total scale at the end of treatment and at follow-up. This demonstrates an important link between illness severity and functional impairment. One explanation for the lack of significance for the social functioning scale at follow-up may be that the CGI relates most well to functional behaviours that are associated with clinical syndromes, such as ADHD, emotional instability and behavioural control.

A strength of the study is the randomized methodology and a reasonable sample size. Additionally the independent raters of the CGI were blinded to treatment condition. The study’s main limitation is the high dropout rate, which left us with a substantially reduced sample at follow-up. Just over half of the sample completed the programme. High attrition has also been reported in other studies [33, 34].The TAU/MED group did not receive any active non-medication control intervention as part of the research protocol, which may also have inflated the treatment effects in the CBT/MED group. Nevertheless, all the participants were receiving medication for ADHD, which in itself should be considered to be an active control. The patients had all been clinically diagnosed with ADHD but from different centres. Participants were assessed by the researchers on the MINI International Neuropsychiatric Interview at baseline, but did not receive any further ADHD clinical assessment. Dosage and treatment compliance were not systematically recorded, although stability on medication was self-reported.

It is notable that participants presented with a high rate of ADHD symptoms—their mean symptom ratings were over 10 points higher than those obtained in the RATE validation studies (19). This suggests that participants were a severely impaired group who may have been poor responders to medication. Hence, the combination of the R&R2ADHD programme with medication significantly improves the treatment effect.

To conclude, the present findings complement our previous findings and together demonstrate that those individuals receiving the multimodal treatment of ADHD medication plus R&R2ADHD will experience a significant reduction in ADHD and comorbid symptoms and that this improvement translates into everyday function. The key mechanism associated with improved functional outcomes is likely to be behavioural control.
